# Reflections from the Lebanese field: “First, heal thyself”

**DOI:** 10.1186/s13031-018-0144-2

**Published:** 2018-02-26

**Authors:** Zeina Chemali, Hannah Smati, Kelsey Johnson, Christina P. C. Borba, Gregory L. Fricchione

**Affiliations:** 1 The Chester M. Pierce, MD Division of Global Psychiatry, Massachusetts General Hospital, 151 Merrimac Street, 4th Floor, Boston, MA 02114 USA; 2 Harvard University, Cambridge, MA 02138 USA; 3 Department of Psychiatry, Boston Medical Center, 1 Boston Medical Center Place, Boston, MA 02118 USA; 4 Department of Psychiatry, Boston University School of Medicine, 72 East Concord Street, Boston, MA 02118 USA; 5 Department of Psychiatry, Massachusetts General Hospital, 55 Fruit Street, Boston, MA 02114 USA; 6 Harvard Medical School, 25 Shattuck Street, Boston, MA 02115 USA

## Abstract

Humanitarian aid workers caring for Syrian refugees face major stressors as they attend to refugees’ needs on the field. Without adequate psychosocial support, evidence has shown that fieldworkers experience high burnout and turnover as well as long-term poor mental health. Unfortunately, scarce training in this regard leaves them ill-equipped to care for themselves and practice resilience while handling trauma in the field. This paper highlights our reflection on working with mindfulness programs during humanitarian crises, specifically how our program, Stress Management and Relaxation Response Training (SMART), has helped over time fieldworkers and the community they cared for. We propose that programs targeting the wellbeing of fieldworkers should be prioritized as part of efforts to improve the international aid response although they may require impeccable coordination and generous resources. We encourage donors to fund those projects viewed as special social protection programs building resilience and strengthening within system support. We argue that this will increase the efficacy of the crisis intervention and work towards sustainable peace building.

## Introduction

In this paper, we will examine field interventions throughout the Syrian refugees crisis in Lebanon starting in 2014. As such, we will analyze two principal subjects: 1) the limited attention to stress management training for fieldworkers despite their work in a space of daily trauma, and 2) evidence that the Stress Management and Resilience Training Relaxation Response and Resilience Program (SMART-3RP) and other mind-body programs could fill in this gap as a public health intervention. By providing context in these areas, we seek to develop potential policy suggestions and next steps to improve the effectiveness of crisis interventions with renewed attention to mental health in the field [1]. Efforts to improve fieldworker mental health will be one critical step in efforts to improve the humanitarian aid response. Beyond resilience training programs like SMART, humanitarian organizations must invest in better preparation and management of their aid workers through structured general training for their work, increased screenings before they are deployed, and upgraded protection measures to ensure their safety in the field. There is no doubt that these steps will require ground coordination, funding, and significant time commitments from donors and international agencies alike, but may move us a step forward towards peace building with optimal host community-refugee population relations and sustainable community interchange. Ultimately, adapting programs like SMART to the Syrian conflict is an important, additional investment in human capital during times of crisis.

## Fieldworkers at risk

Aid workers in humanitarian crises face tremendous challenges in facilitating relief and resettlement [2, 3]. Even after the acuteness of the crisis has abated, international agencies often leave the terrain to local and national groups to attend to the day-to-day hardships of caring for victims [15]. Within Syria and in host countries like Lebanon and Jordan, the humanitarian force is stretched thin in assisting millions of refugees. Despite evidence of psychological stress and physical danger, trauma amongst health workers is often put last or unaddressed entirely. Adequate self-care training and mental health support services are essential to destigmatize worker vulnerability and promote wellbeing within the humanitarian force in the Syrian conflict [4].

### Culture of trauma

Relief workers in international conflicts are susceptible to both primary and secondary trauma through their work, and are increasingly becoming targets of violent attacks, abductions, and other tactics meant to intimidate local populations and foreign intervention [1, 5]. Since the start of the Syrian war in 2011, this violence has resurged and hit a breaking point with over 650 medical personnel killed [6]. In 2016 alone, the WHO reported confirmed reports of 338 attacks on health care facilities across Syria [15]. Humanitarian workers have been reported to be more likely to die of intentional violence than accidents or even coincidental illness [7].

Although there is still a dearth of research into trauma and mental health issues among staff in today’s humanitarian crises, available literature reveals a disproportionate risk of depression, anxiety, and especially posttraumatic stress disorder (PTSD) [1]. This mental health crisis could result in unhealthy behaviors to deal with trauma, including the documented reliance on potentially risky behaviors and increased alcohol consumption. One study found significantly heightened levels of heavy drinking amongst aid workers in the Middle East region, closely associated with overcommitment and work stressors [8]. Aid workers experience significant symptoms of burnout, and there is disproportionately high staff turnover across non-governmental organizations [2, 9, 10].

These gravely poor health outcomes could endanger not just those entering the aid workforce, but also disrupt the efficacy of larger coordinated humanitarian operations.

### Stigma without support

Despite the overwhelming prevalence of mental health problems, there still exists a stigma in discussing vulnerability or trauma. In a survey of aid workers conducted by The Guardian, interviewees reported a “culture of silence,” with workers feeling self-conscious when speaking up about their mental issues for fear of not seeming like a “true humanitarian” [11]. Even in the first stages of the Syrian conflict, fieldworkers were given little time or space to process the traumatic events of the day, feeling too embarrassed to discuss their vulnerabilities for fear of coming off as too emotional [4]. This unspoken stigma is exacerbated by the lack of a standardized mental health support service for aid workers, particularly as international organizations defer to local and national groups to run daily operations.

Studies have shown the significant value of psychological support for staff at the level of their organization. In one study, Sri Lankan humanitarian workers who reported high levels of support from their organizations were less at risk for symptoms of depression and PTSD [12]. This support system ought to be present at all stages—before, during, or after deployment—in order to improve mental health outcomes. Inadequately preparing or supporting fieldworkers does not just leave them more vulnerable to trauma and stress, but it also encourages further negative feelings both towards the self and towards the organization. Without mindful support mechanisms in place, aid workers could be at risk for feelings of low self-esteem, disappointment and anger with the organization, or lack of achievement regarding one’s purpose [13]. Though there have been calls for stress management training and psychosocial support services based on these findings, there is still a paucity of in-crisis interventions or a standardized strategy for pre-deployment preparation for potential work stressors [5, 14].

### Need for psychosocial support in Syria

The complexity of the Syrian conflict puts humanitarian staff at a uniquely acute risk for trauma-related mental health problems. There were reported to be nearly 5 million registered refugees in Turkey, Lebanon, Jordan, Iraq, and Egypt by the end of 2016, with over 200 NGOs and agencies working tirelessly to provide medical care, food, water, and protection [15]. Community workers must not only work on the ground to coordinate this work, but must do so while navigating the often tense interactions between refugee communities and host countries. The high refugee density in the region has stressed public resource allocation, and local economic challenges have curbed host government’s abilities to respond [16]. To mediate this burden of resettlement and bolster the effectiveness of international aid, it is essential to foster a renewed focus on social protection and investment in human resources, namely health workers [17]. Especially in light of the long-term and violent nature of the Syrian conflict, there must be renewed attention on resilience and self-care through psychosocial training and support for aid workers.

## Training in mindfulness and self-care

Earlier this year, our group published our work in developing a psychosocial training intervention to improve mental health outcomes of Lebanese fieldworkers during the Syrian refugee crisis. We translated and adapted the SMART-3RP program, developed by the Benson-Henry Institute for Mind Body Medicine at Massachusetts General Hospital [4]. SMART- 3RP trains workers to deal with chronic stress and has been shown to improve perceptions of daily stress, reduce depressive symptoms, foster empathy, and improve health-related quality of life in addition to physiological and transcriptomic benefits [18, 19].

### Stress management and resilience training relaxation response and resilience program (SMART-3RP) overview and scientific basis

The SMART-3RP blends techniques in stress management, cognitive behavioral therapy, and positive psychology. It centers on coping and increasing resiliency capabilities by teaching a relaxation response (RR) [20]. The RR is the inverse of the stress response, defined as decreased arousal of the sympathetic system [21]. Throughout the program, participants learn better coping strategies that mitigate the physiological and cognitive perceptions of the stress response [20]. The end goal is to teach better self-care, providing strategies to adapt and cope with daily stressors.

Though it is not a treatment for depression, the mind-body program has been shown to significantly decrease depressive symptoms [22]. Improved physiological markers associated with stress, such as lower blood pressure and enhanced mitochondrial resiliency, have also been linked [4, 19, 23, 24]. The program has also been shown to be effective in managing symptoms of PTSD and anxiety and in improving resiliency among U.S. military veterans, older adults, palliative care clinicians, and adults with tumor suppressor syndromes [25–28].

### Better outcomes for Lebanese fieldworkers

In 2014, our group translated the SMART training program into Arabic and adapted it to suit the challenges of aid workers, consulting The Syrian Project Desk at the Lebanese Ministry of Social Affairs (MOSA) for cultural appropriateness and wording. Social workers were recruited from regions across Lebanon through flyers prepared and submitted by the MOSA. The recruitment was open to all social workers interested in the program with priority given to social and fieldworkers involved with Syrian refugees. One hundred twenty participants enlisted and all underwent the training. Of the 120 fieldworkers who underwent SMART training, 100 opted to participate in the survey portion of our research with 20 abstaining from enrolling in the study and their reservations were noted. Our results noted that the SMART program was helpful in encouraging mindfulness and self-reflection. When applied to their stressful environments, staff reported that these practices allowed them to be more aware of their negative emotions and think more positively. They also voiced improved problem-solving capabilities, with greater patience in overcoming challenging situations [4].

The survey responses from the SMART-3RP trained humanitarian workers in Lebanon also highlighted interpersonal skills that developed in parallel with personal stress management—a valuable capacity for working with such a large and vulnerable refugee population. Participants reflected that thinking about their own emotions helped them better understand other refugees, and also that they intended to use their SMART strategies to show empathy [4]. Literature has highlighted the potential for positive emotions like compassion and sympathy to be an emotional coping strategy for reducing the magnitude of a stressful reaction to an event and indirectly improving physical health outcomes [29, 30]. Consequently, mind-body training could be used as valuable leverage to strengthen trust and connections with local communities as well as fieldworkers’ resilience, health, and communication skills. As workers build long-term relationships with resettled refugees and help with integration into host populations, these interpersonal skills and self-care will be crucial to the success of their mission.

### Scaling up to remedy a paucity of support

As previously discussed, there is a significant lack of psychosocial support mechanisms for aid workers in the field. Few organizations report hands-on training of their personnel in stress management, or screen their staff for potential risk factors in stress responses [31]. Despite this paucity, it is clear that perceived inadequate support can negatively impact a worker’s motivation and thinking, and adequate preparation on the organization level can reduce risk of depression and anxiety [13, 31]. In line with this literature, the workers in Lebanon who underwent SMART-3RP training gave positive feedback and expressed wishes that it be made accessible to others and to continue the training, even calling it a “necessity” [4]. Like formal social protection programs, empathy and resilience training programs could take on a protective capacity and improve the adaptability of fieldworkers, systems, and refugees alike, all while facilitating better ground coordination between different humanitarian international, local agencies, and global donors [32]. Having said that, we would like to note that the facilitation and communication on the ground should be also reinforced and supported at a leadership level by the multinational humanitarian organizations.

## Conclusion

As the Syrian conflict enters its seventh year, it is essential that the international response puts new support mechanisms in place for fieldworkers, and fundraises for self-care and resilience programs. Stress management training represents an alternative way to improve wellbeing and enable better communication and understanding in host community-refugee interactions. There is no doubt that humanitarian workers continue to be frequently targeted during conflicts [33]. They are stretched to their limit providing care for a large vulnerable population while experiencing secondary trauma from their interactions. For that reason alone, the international agencies must redouble their efforts to improve their personnel training to encompass the traumatic events they will face in the field. Programs like SMART-3RP are one important part of this effort.

Our reflections from the field in Lebanon following the Syrian refugee crisis highlight the tremendous psychosocial burden put on humanitarian workers, and invite the crisis intervention community to reinvest both in fieldworkers’ well-being and the resilience of host and refugee communities. Delivering trainings in mindfulness and self-care such as SMART-3RP would encourage and protect the quality of life for all in the field. As humanitarian missions scale up, this new resource would be a valuable addition to known manuals and workshops in the humanitarian field. We invite the field to open the debate and facilitate research to study those outcomes in depth.

## Abbreviations

MOSA: Lebanese Ministry of Social Affairs
NGO: Non-government organization
PTSD: Posttraumatic stress disorder
RR: Relaxation response
SMART-3RP: Stress Management and Resilience Training Relaxation Response and Resilience Program
WHO: World Health Organization
